# Management of the mangled extremity

**DOI:** 10.1007/s11751-012-0137-4

**Published:** 2012-06-13

**Authors:** Mark L. Prasarn, David L. Helfet, Peter Kloen

**Affiliations:** 1 Department of Orthopaedic Surgery, University of Texas Medical School at Houston, Houston, TX USA; 2 Hospital for Special Surgery, New York, NY USA; 3 Department of Orthopaedic Surgery, Academic Medical Center, G4-N, Meibergdreef 9, 1100 DD Amsterdam, The Netherlands

**Keywords:** Mangled extremity, Limb salvage, MESS, Fracture

## Abstract

The management of a mangled extremity continues to be a matter of debate. With modern advances in trauma resuscitation, microvascular tissue transfer, and fracture fixation, severe traumatic extremity injuries that would historically have been amputated are often salvaged. Even if preserving a mangled limb is a technical possibility, the question is often raised whether the end result will also be functional and what treatment would lead to the best patient outcome. The road to salvage is often prolonged with significant morbidity, reoperations, financial costs, and even mortality in some instances. Numerous factors have been implicated in the outcome of these injuries, and a number of scoring systems have been designed in an attempt to help guide the treating surgeon in the acute phase. However, much controversy remains on the ability of these grading systems to predict successful salvage of the mangled extremity. In this review, we discuss the mechanisms of injury, various available scoring systems, initial management, outcome and specific differences between lower and upper extremity trauma injuries.

## Introduction

The definition of a mangled extremity is a limb with an injury to at least three out of four systems (soft tissue, bone, nerves, and vessels). Mangled extremities have historically been associated with very high amputation rates. Advances in evacuation, resuscitation, wound care, free tissue transfer, and internal fixation make it nowadays possible to salvage limbs that would have been amputated in the past. Experience based on these injuries from a combat setting in World War II, the Korean and Vietnam War, and more recently in the Middle East (Operation Enduring Freedom and Operation Iraqi Freedom) has shown clearly progress with amputation rates for mangled extremities is decreasing from 72 to 13–20 % to less than 10 %, respectively [1]. Despite these technical advances, the management of a mangled extremity remains a very difficult decision process for the patient, his/her family, and the treating surgical team. Moreover, the mangled extremity is often the result of a high energy trauma that will have caused severe injuries to other organ systems (brain, chest, and pelvis) as well. Resuscitation and management of all life-threatening injuries always must take precedence over any extremity injury (life before limb), so that definitive treatment of the mangled extremity (other than primary amputation) is seldom indicated in the acute phase.

In patients with complete traumatic disruption and clearly irreparable injuries, an immediate completion amputation should be performed (this is a very small subset). Likewise, in the setting of prolonged limb ischemia, severe soft-tissue loss that cannot be reconstructed or concurrent life-threatening injuries in an unstable polytrauma patient, a primary amputation is indicated. Although the decision to amputate in the acute setting is difficult for the patient, family, and the treating surgical team, the alternative of prolonged unsuccessful attempts at limb salvage will subject the patient to great physical, psychologic, financial, and social suffering [2]. This leaves the majority of mangled extremities as potentially salvageable for which, in the acute setting, a treatment plan needs to be made.

In this review, we will present an overview of the current controversies and outcome data available.

## Mechanism of injury

The majority of mangled extremities are due to blunt trauma. Motor vehicle crashes and industrial/farm accidents are the leading causes of such injuries in both the upper and lower extremities [3–7]. Falls from a height, high-velocity gunshots, and explosion injuries constitute the remainder of mechanisms [8]. Increasingly a specific subgroup being described is based on combat (blast) injuries sustained in the Middle East [1]. The most significant factor involved with the injury mechanism is the amount of energy transferred to the extremity rather than the actual mechanism. The relative amount of energy absorbed directly translates into the amount of destruction to the bone and soft tissues. The term “zone of injury” has been coined to define the area of the extremity affected by the injuring force. This zone may be defined by the fracture type, the amount of comminution, the area of crush, laceration, or shearing of the soft tissues, or devascularization of the entire limb [9].

## Initial management

Initial management of the patient with a mangled extremity begins with ATLS protocol emphasizing a primary survey with immediate assessment of the ABC’s. Following this, the field dressing should be removed and any significant bleeding immediately controlled with direct pressure, tourniquet, a compressive dressing, or proximal clamping (in that order of preference). Exploring the wound in the Emergency Room is not advantageous, as this can precipitate further bleeding and lead to further wound contamination.

Once the resuscitative effort is underway, further assessment of other injuries should be undertaken as well as a thorough neurovascular examination. Injuries that are associated highly with vascular compromise are supracondylar femur fractures, knee dislocations, proximal tibia fractures, and penetrating injuries of the posterior and medial thigh. If there is disruption to the arterial flow to the extremity, and salvage is being considered, an intraluminal shunt should be used. Warm ischemia time should not exceed 6 h for the lower extremity and 8 h for the upper extremity. The site of vascular injury can often be deduced from the fracture pattern and critical time should not be lost on vascular studies in the radiology suite. Wound dressing, gross alignment, and splinting should be performed. Following this, any radiographic studies may be obtained (including vascular studies if necessary), and intravenous antibiotic and tetanus prophylaxis administered. A Mangled Extremity Severity Score (MESS) is calculated for each patient at the onset of treatment [1]. If an early amputation is deemed necessary it is often advantageous to take medical record photographs to document the severity of the injury. We also recommend keeping a photographic record throughout the course of treatment if reconstruction is performed, to document both progress and decline.

Our indications for early amputation include unreconstructable osseous or soft-tissue injuries, irreparable vascular injuries, and severe loss of the plantar skin and soft tissues. Previous authors have recommended amputation if plantar sensation is absent. Bosse et al. [10] have suggested that initially absent plantar sensation does not predict a poor functional outcome and that it may return in more than half of patients followed out to 24 months. We do not use absent plantar sensation as a sole criterion for a primary amputation. Lange stated in his classic article in 1989 that most patients will not have an absolute indication for amputation but will fall into an indeterminate gray zone [11]. His absolute indications for amputation were anatomic nerve disruption, warm ischemia time > 6 h, ipsilateral mangled foot, and hemodynamic instability. Even though this article is over 20 years old, the only absolute indication that would now be disputed would be nerve disruption. One would have to perform significant dissection in the zone of injury to confirm nerve transection, and this is not typically done as it causes significant additional soft-tissue damage. As stated above, loss of plantar sensation alone does not necessary indicate nerve disruption and is not an appropriate indication for amputation [10].

**Fig. 1:**
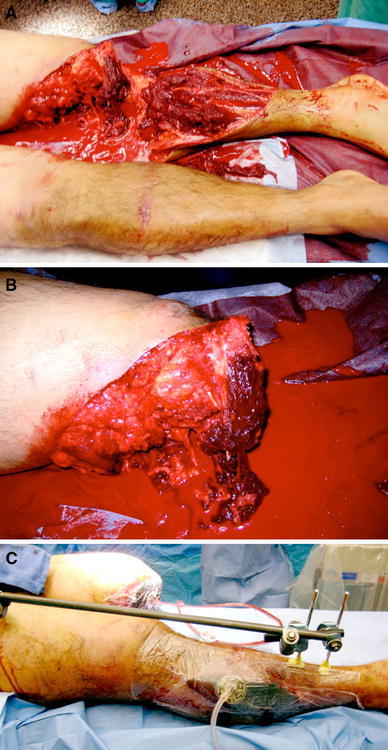
A 21-year-old male presented to the emergency department following a motorcycle collision with bilateral lower extremity injuries. **a** Left-sided pulse-less (Grade IIIC) “mangled” knee/lower extremity injuries and a right-sided bicondylar closed tibial plateau fracture with compartment syndrome (*top image*). **b** Left-sided completion of the above knee amputation retaining as much viable soft tissue as possible (*middle image*). **c** Application of negative-pressure wound therapy dressing to left-sided amputation site, as well as external fixation of right bicondylar tibial plateau fracture and leg fasciotomies for compartment syndrome (*bottom image*)

The amputation should be performed at the most distal level possible but should not include clearly nonviable tissues. Color, consistency, contractility, and bleeding of the soft tissues should be used to determine viability. It has been shown that transtibial amputations have significantly better functional outcomes and lower energy expenditure than more proximal levels of amputation [9, 12]. A thorough irrigation and debridement should be performed without any attempt to close the wound at this time. A sterile dressing or wound negative-pressure dressing can be applied, and a splint applied if the amputation is below the level of the knee or elbow (Fig. 1). Return to the operating room with repeat surgical debridement should be performed as deemed necessary. In most instances, several irrigation and debridements are undertaken prior to closure of the stump site. Debridements should be performed by—or under direct supervision of—a senior experienced surgeon. This part of a limb salvage should not be taken lightly. Although there a no data on this issue, we think that debridements by surgeons-in-training are generally more conservative than those done by senior surgeons.

If the need for amputation is not clear upon initial examination, then limb salvage should be attempted. Once again, a thorough irrigation and debridement with removal of any contaminants and nonviable tissue performed emergently. In this acute phase, damage control orthopedics (DCO) with temporizing measures (external fixation, fasciotomies, temporary shunting) has been shown to be effective, straightforward and quick [13, 14]. If necessary, a definitive vascular repair should be performed following skeletal stabilization. Ex-fix pins should be placed strategically away from the zone of injury and based on future incisions for definitive ORIF. Compromise of formal ORIF after DCO using external fixation is generally not an issue [15]. Antibiotic bead pouches and negative-pressure wound therapy (VAC) can be used to help decrease infection and assist with wound care [16, 17]. The extremity is closely monitored over the next 2–3 days for soft-tissue viability and sensorimotor function. Wounds should be regularly inspected and repeat irrigation and debridements performed based on wound appearance (tissue viability, presence of contaminants, infection, etc.). Negative-pressure dressings are changed every 48–72 h.

**Fig. 2:**
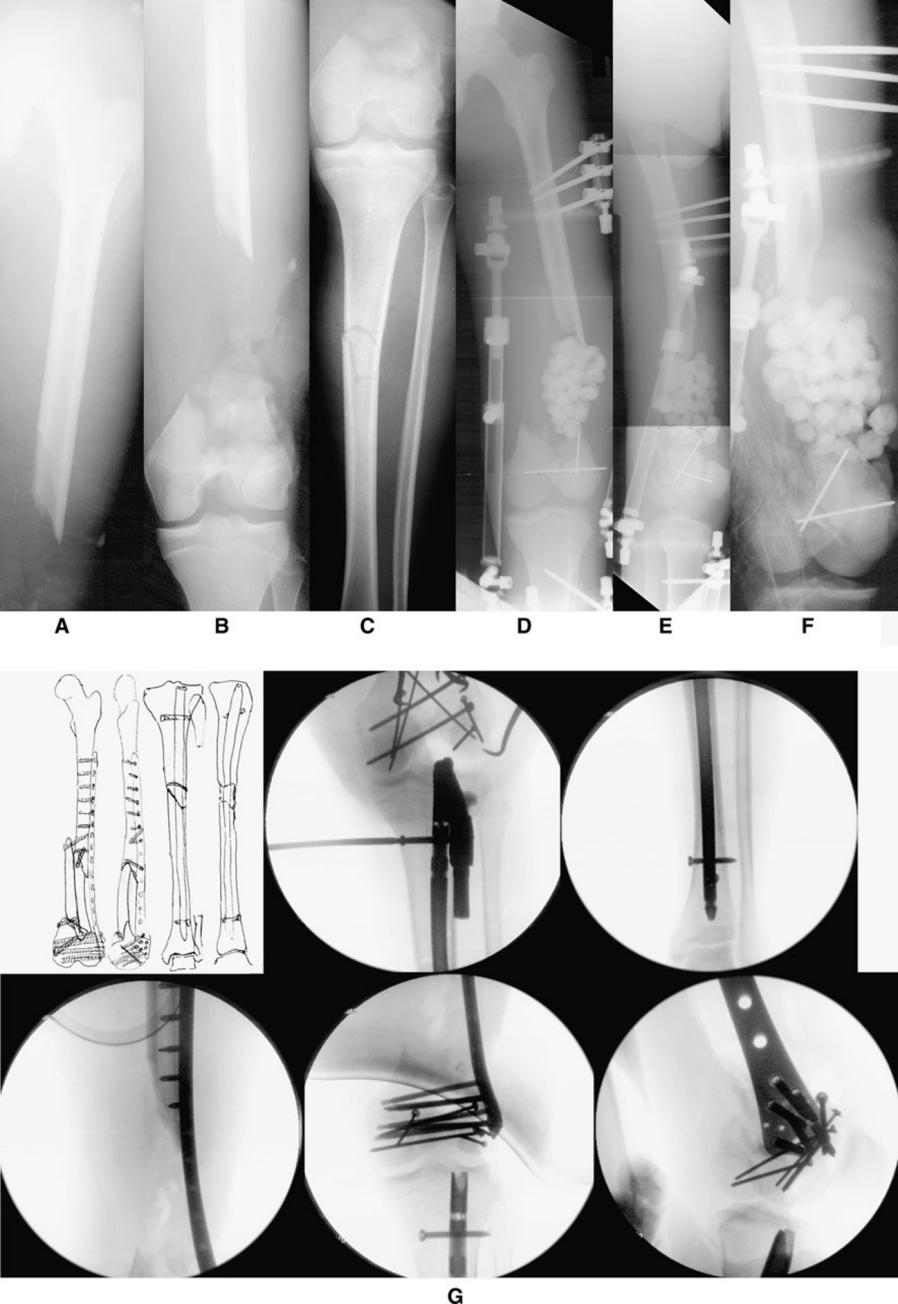

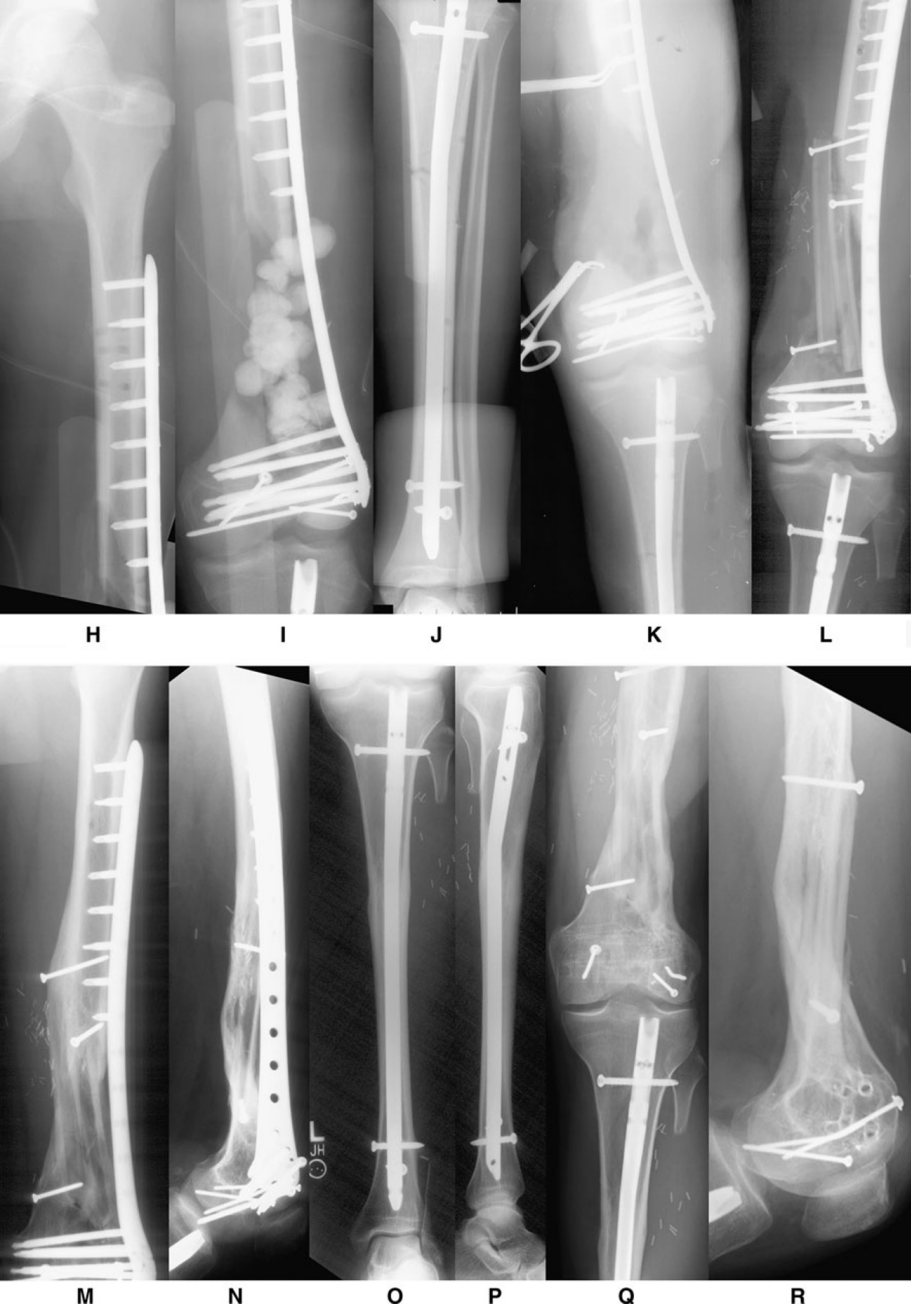
A 17-year-old male was involved in a head-on collision with a tractor trailer. After being trapped inside the vehicle for approximately one hour, he was extricated and flown to a local trauma center. He was diagnosed with an open, Grade IIIC left-sided AO/OTA Type C3.3 distal femur fracture with segmental defect and an ipsilateral tibial shaft fracture. External fixation was placed for initial stabilization, and antibiotic beads were subsequently placed in the defect at 3 days following injury. Open reduction and internal fixation (ORIF) was performed with placement of an intramedullary (IM) locked nail for treatment of the tibial shaft fracture and then ORIF of the distal femur fracture with placement of a less invasive stabilization system (LISS) locking plate and screws. One week later, the antibiotic beads were removed and the defect was prepared for bone graft placement. A second incision was made along the lateral border of the ipsilateral fibula, and a free vascularized fibula bone graft was harvested for transplant to the femoral defect. It was docked in a double barrel fashion and stabilized using screw fixation. Following surgery, he returned for regular follow-up visits. Three months after surgery, all of the fractures were healing with incorporation of bone graft. The LISS plate was removed 4.5 years following the initial surgery. The clinical and radiographic follow-up illustrated excellent results with bony union, full range of motion, and complete resolution of pain and return to preinjury activities. **a**, **b**, **c** Anteroposterior (AP) x-rays illustrating an AO/OTA Type C3.3 distal femur fracture with segmental bone defect and an ipsilateral tibial shaft fracture. **d**, **e**, **f** AP and lateral radiographs following placement of external fixation and antibiotic beads at the site of the segmental bone defect. **g** Clockwise from top-left; preoperative plan, fluoroscopic images showing placement of intramedullary nail for the tibial shaft fracture and locking screws and ORIF of the distal femur fracture with placement of a LISS locking plate and screws. **h**, **i**, **j** Immediate postoperative radiographs demonstrating adequate fixation and alignment. **k** AP radiographs illustrating preparation of distal femoral bone defect for placement of vascular bone graft. **l** AP x-radiograph following free vascularized fibular bone and placement of screw fixation. **m**, **n**, **o**, **p** AP and lateral x-rays 3.5 years following ORIF showing healed a distal femur fracture with incorporation of the fibular bone graft and a healed tibial shaft fracture. **q**, **r** AP and lateral x-rays 8 months following removal of LISS plate and screws and 4.5 years following fracture surgery

If at any point the limb is deemed unsalvageable or the patient’s life in jeopardy secondary to the extremity, an amputation should be performed. If the extremity remains viable for reconstruction and the patient condition permits then definitive skeletal stabilization and early soft-tissue coverage can be performed [18]. Various modalities are available for surgical fixation including uniplanar external fixators, hybrid external fixators, thin-wire ring external fixators, plate and screw constructs, and intramedullary nails (Fig. 2). The specifics of bone and soft-tissue reconstruction are beyond the scope of this review.

## Scoring systems

Multiple scoring systems have been proposed by various authors to help guide the management of complex extremity trauma. Even so, there is still much debate over the criteria that can help in predicting limbs that can be successfully reconstructed and ones are better off with early amputation [19–21]. Most of these predictive indices have been criticized as being too subjective, complex, and difficult to apply universally. Most are derived retrospectively from small patient series and not validated with functional outcome data [9, 22]. We will briefly discuss their pertinent findings and shortcomings.

The Predictive Salvage Index (PSI) was developed by Howe et al. [23] in 1987 to use in the setting of combined orthopedic and vascular lower extremity injuries. Points are assigned for the level of arterial injury, degree of bone and muscle injury, and the time elapsed from injury to arrival to the operating room. In a small, retrospective analysis of 21 patients, all 12 patients with successful limb salvage had a PSI < 8, while 7 of the 9 who underwent amputation had a PSI of at least 8 (sensitivity of 78 % and specificity of 100 % for predicting amputation). Other authors have reported much lower sensitivity and specificity of the PSI [22, 24].

The Mangled Extremity Severity Score (MESS) was introduced by Johansen et al. [20] in 1990 based on a retrospective review of 26 mangled lower limbs in civilian practice. Four different factors are scored: skeletal and soft-tissue injury, ischemia, shock, and patient age. The scores are summated to a maximum of 15. A value of < 7 was shown to be predictive of salvage [19, 20]. The proposed advantages of this predictive index are that the information is readily available upon presentation, its relative simplicity, and reproducibility. Rush et al. [1] showed in a combat setting the MESS was a sensitive predictor of amputation. In contrast, a larger study by Brown et al. [8] in British military patients (Iraq and Afghanistan conflicts) with mangled extremity ballistic injuries found that the MESS did not help decide whether or not an amputation was appropriate. Others have criticized the subjectivity of the MESS, and review of larger series of patients has shown lower sensitivity of the index than initially reported [22, 25–28].

One year later, in 1991, Russell et al. [21] proposed the Limb Salvage Index (LSI) based on the review of 70 limb-threatening injuries. The index predicts the likelihood of limb salvage based on ischemia time and injury severity to six types of tissue that may be involved. The score can only be assigned after extensive examination during an operation and is a useful score in the decision-making process.

The Nerve injury, Ischemia, Soft-tissue contamination, Skeletal injury, Shock and Age (NISSA) was introduced by McNamara et al. [28] in 1994. This system is a more complex modification of the MESS that separates the skeletal and soft-tissue injury and adds a nerve injury component. In a small retrospective series (24 patients), the authors concluded that the system is more sensitive and specific than the MESS. It has been criticized for placing too much emphasis on loss of plantar sensation in the acute phase as this is often a crush neurapraxia that resolves over time [29].

Two studies have examined the ability of these scoring systems to predict functional outcome following treatment [30, 31]. Both showed no significant differences between patients with good or poor functional outcomes, and none of the scoring systems analysed were able to determine outcome. Based on these two studies, it seems the commonly applied predictive indices may be useful in early decision-making but are unable to predict functional recovery. The treating surgeon and patient still have no objective simple criteria to assist in making such a monumental decision.

## Complications

A major factor in the decision-making in the treatment of the mangled extremity is the risk of major complications in each treatment arm. Great insights are provided by the Lower Extremity Assessment Project (LEAP) funded by the National Institutes of Health. In this study, a cohort of 545 patients with severe lower extremity injuries was followed prospectively for 24 months. Eight level I trauma studies participated in this investigation. A physician examined each patient at 3-, 6-, 12-, 24-month intervals and major complications recorded. Harris et al. [32] reported the nature and incidence of major complications for this cohort. The two most common complications were wound infection (28.3 %) and nonunion (23.7 %), most of which required operative intervention and/or inpatient care. Approximately a quarter of each of these complications were considered severe enough to compromise long-term function. The overall incidence of osteomyelitis was 7.7 %.

The complication data from the cohort were further examined based on treatment arm in the study. In the *limb reconstruction* group (*n* = 371), the most common complication was nonunion (31.5 %), followed by wound infection (23.2 %). Of these infections, 8.6 % developed into osteomyelitis. There was an incidence of post-traumatic arthrosis of 9.4 % and wound necrosis or breakdown of 6.5 %. A total of 149 patients underwent *amputations,* and the revision amputation rate was 5.4 %. The most common complications in this group were wound infection (34.2 %), followed by stump revision (14.5 %), phantom limb pain and wound breakdown (13.4 % each), and stump complications (10.7 %). The *late amputation* group (patients amputated after initial discharge) experienced the highest rate of major complications (85 %). Most common complication in this group was infection (68 %), osteomyelitis (40 %), and stump complications (24 %).

Bondurant et al. [2] undertook an investigation looking at the effects of delayed versus primary amputation. There was a significant increase in length of hospital stay (22 vs. 53 days) and number of surgical interventions (1.6 vs. 6.9) when comparing early versus delayed amputation, respectively. The cost was almost double, and there was a 21 % mortality rate in the delayed amputation group. It is quite evident that every effort should be made to avoid a late amputation given such high costs for all involved.

In a prospective cohort study (using LEAP study patients), Castillo et al. [33] examined the specific effect of smoking on complication rate in 268 severe open tibia fractures. Nonunion rates were significantly higher in both the current and previous smoking groups (37 and 32 %, respectively). Current smokers were twice as likely to develop an infection and 3.7 times more likely to have osteomyelitis. Previous smokers were 2.8 times as likely to develop osteomyelitis as nonsmokers [33].

## Outcomes following limb salvage versus amputation

Medical and surgical progress has dramatically improved our ability to salvage severely injured extremities. Limbs that historically would have been amputated can now often be managed with complex reconstruction techniques. This might come with a price of years of hospitalization time, multiple surgeries, complications, and for some an inevitable amputation. For these secondary amputations, it is often questioned whether or not the patient would have been better served with a primary amputation. Limb salvage patients often still complain of edema, pain, decreased sensation, difficulty with footwear, and ambulation. The end result can be a physical, psychologic, financial, and social cripple with a useless salvaged limb [34]. Even so, cultural and religious concerns and differences vary throughout the world. In developing countries, an amputation is often not considered an option by the patient and his/her family, who consider a limb with continuing problems superior to an amputation.

Hoogendorn and van der Werken [34] looked at the long-term outcome (according to AMA impairment guidelines) and quality of life (using SF-36 and the Nottingham Health Profile) of patients treated with reconstruction versus amputation following Grade III open tibia fractures. A total of 64 patients were assessed, including 43 with successful limb salvage and 21 who underwent amputations (including both primary and delayed). Patients who underwent amputations had more severe injuries and had a higher number of vascular injuries (77 vs. 17 %). The limb salvage group underwent more operations and had more complications. Delayed amputations were performed in 8 patients, most commonly because of persistent infection and poor soft tissues. They were hospitalized twice as long as those who underwent primary amputation. Others have also shown that delayed amputation results in poorer functional outcome versus primary amputation [2, 35]. From the reported health surveys, the authors found low scores in both groups but no significant differences. In both groups, over half the patients considered themselves disabled, with a slightly higher percentage of patients who had amputations reporting difficulty with practicing a profession (60 vs. 40 %). Of particular interest was that the mean lower extremity impairment score was significantly worse for amputees (73.5 %) as compared to the limb salvage group (17.6 %) [34].

The LEAP study group examined the functional outcome using the sickness impact profile (SIP) following limb salvage versus amputation with a follow-up of 24 months for 84.4 % of the patients. Comparisons of outcomes for the SIP were adjusted for potential confounding variables of the patient characteristics as well as their specific injuries [4]. It was noted that patients who underwent amputation had more severe injuries, but otherwise did not differ from those who had reconstruction [4, 36].

Upon examining final functional outcome, there were no significant differences in scores between either treatment groups, although 42 % of the patients had scores greater than 10 indicating severe disability. Patients who underwent limb salvage were more likely to have been re-hospitalized than those who had amputation performed (47.6 vs. 33.9 %, *p* = 0.002). Multivariate analysis reveals several factors that were significant factors for a poor outcome including: re-hospitalization for a major complication, having less than a high-school education, low household income, having no insurance or Medicaid, being non-white, smoking, having a poor social-support network, having a low-level of self-efficacy, and being involved with the legal system for injury compensation. At final follow-up, approximately 50 % of patients had returned to work and this rate did not differ between the two groups [4].

Patients with bilateral mangled extremities were excluded from the initial above analysis in the LEAP study but were followed prospectively and reported on separately. There were a total of 32 bilateral injuries, of which 14 had bilateral salvage, 10 had bilateral amputation, and 8 had unilateral salvage/amputation. Forty-six percent of patients were severely disabled at the 24-month follow-up as demonstrated by SIP scores > 10. Once again, the groups where salvage procedures were performed had higher re-hospitalization rates for complications than the bilateral amputation group. The return to work rate was higher in the unilateral amputation/salvage group, and they had faster walking speeds. Examination of all three combinations of treatment of bilateral limb-threatening injuries in the LEAP study (*n* = 32 patients) demonstrated similar outcomes at 2 years. The evidence from this study suggested that the disability for bilateral limb-threatening injuries is high, but no more so than the unilateral group described above. The authors therefore concluded that treatment strategies for bilateral mangled extremities should be derived from the results from the larger cohort study of unilateral injuries [37].

MacKenzie et al. [12] reported on the long-term follow-up of the original patients included in the LEAP study. The main goals of the study were to determine whether the previously reported outcomes improved after 2 years, and whether there were any late differences between the treatment groups. Of the 569 patients from the original cohort, 397 were contacted by phone at an average of 84 months post-injury (range, 70–90 months). On average, most of the patients reported physical and psychosocial functioning that had deteriorated since their 24-month follow-up (*p* < 0.05). This increase in SIP scores was consistent across both treatment groups. It should be noted thought that patients who underwent through knee amputations were at the highest risk for a poor outcome. More than a third of patients in both groups had been re-hospitalized between 2 and 7 years post-injury. At final follow-up, almost 50 % of the patients indicated severe disability, with SIP scores > 10. Only 34.5 % of the cohort had a physical SIP sub-score typical of the general population (< 5).

The Evidence-Based Orthopaedic Trauma Working Group performed a meta-analysis of observational studies on complex limb salvage or early amputation for severe lower-limb injury. They found no significant differences in functional outcome at least up to 7 years [38]. A recent meta-analysis evaluating the quality of life (measured with SF-36 and SIP) in post-traumatic amputees (769 patients) in comparison with limb salvage (369 patients) showed that limb salvage in a mangled extremity yields better psychologic outcomes compared to amputation even though the physical outcome was more or less the same [39].

## The mangled upper extremity

There are some important differences between the mangled upper and mangled lower extremity, which must be carefully considered by the treating surgeon. Critical time for reperfusion is longer in the upper (8–10 h) versus the lower extremity (6 h) [7]. A transtibial amputation carries a much better functional prognosis than a transradial amputation. This is due to the fact that upper extremity prostheses do not work as well as lower extremity prostheses. Shortening of the humerus to reduce soft-tissue defects is tolerated well up to 5 cm, in contrast to the lower extremity that does not tolerate shortening of more than 2 cm. Nerve reconstruction in the upper extremity is done with reasonable success, whereas in the lower extremity, many consider major nerve injury an indication for primary amputation. The rehabilitation process is also more imperative when the upper extremity is involved [5]. One consistency to both is that the MESS has been shown to be useful for predicting amputation following mangled upper extremities [40].

## The mangled extremity and polytrauma

The question whether amputation of a mangled limb is advisable for a severely injured (polytrauma) patient cannot be easily answered [41]. There are no clear guidelines with respect to the *isolated* mangled extremities, let alone those in the polytrauma patient. An undisputed rule in polytrauma is “life before limb”; meaning life-threatening issues are always addressed first. Orthopedic efforts in the initial resuscitation of the severely injured patient with extremity injury often involve damage control orthopedics (DCO) [14, 15]. DCO polytrauma patients are typically categorized into stable, borderline, unstable, and *in extremis*. The goal of DCO is to minimize subsequent stresses after the first hit (initial injury), and its effectiveness in the context of polytrauma patients with major orthopedic fractures has been shown [15].

As an exception and utilizing DCO guidelines, salvage of the *stable* polytrauma patient’s mangled limb is possibly the most relevant. For these, techniques involving early free tissue transfer and internal fixation as proposed by the “fix-and-flap” technique might be successful but require a highly specialized trauma center with microsurgical expertise [42]. Still, for these patients, the decision to salvage or amputate produces the same dilemmas as with the patient with the isolated mangled limb.

*Borderline* patients that stabilize after resuscitation can undergo early total care (ETC.), but reconstructive efforts may be complicated by potential deterioration. Long procedures (e.g., “fix-and-flap”) are not justified in these patients. Wound debridement, revascularization, and simple external fixation are all that can be done while a rapid turn for the worse should be anticipated. In the *unstable* or *in extremis* polytrauma patient, there might be a role for primary amputation as prolonged revascularization and stabilization procedures add to the patient’s catabolic state and will increase the second hit enormously. Any other reconstructive efforts for the extremities are not justified in the acute stages.

Subsequent surgical procedures for limb salvage should not be undertaken until the patient has stabilized and is beyond the systemic inflammatory response syndrome (SIRS) stage. In general, this means that timing of second and subsequent major procedures (> 3 h surgical time) should be at least after 4 days [43]. If the limb develops evidence of sepsis, early amputation should still be considered. The use of fresh warm blood, plasma, and recombinant factor VII (defined as damage control resuscitation—DCR) will help optimize the physiologic parameters and can, theoretically, allow for more prolonged surgical procedures such as revascularization [44]. DCR may thus provide a means to aid in limb salvage.

## Conclusions

The combination of osseous, vascular, soft-tissue, and nerve injury after severe trauma to an extremity are a great challenge. There is a hierarchy of importance of injuries to each of the four systems in the limb in the following order: soft tissue, nerve, bone, and artery [45]. Unfortunately, the data regarding the management of the mangled extremity are conflicting, and the literature is without Class I studies. It is imperative an experienced surgical team at a trauma center (that sees such patients with some regularity) cares for the patient with a complex extremity injury. The treating team must always keep in mind the high prevalence of associated multisystem trauma and systemic problems related to these injuries. Even though the treatment goal is limb salvage, it must be kept in mind that in many instances, a primary amputation might provide the best outcome. New insights, therapies and techniques will improve outcomes in even the most severely injured patients with complex extremity injuries, but salvage is no guarantee of functionality. As for the mangled limb in these patients, it is unlikely a scoring system will allow a clear cut-off point for amputation versus salvage. What has become clear is that amputation should not be considered a treatment failure but rather a means of meeting goals of treatment.
